# Editorial: New Technologies and Rehabilitation in Neurodevelopment

**DOI:** 10.3389/fpsyg.2022.849888

**Published:** 2022-04-26

**Authors:** Rosa Angela Fabio, Tindara Caprì, Barbara Colombo, Nasrin Mohammadhasani

**Affiliations:** ^1^Department of Economics, University of Messina, Via Verdi 75, Messina, Italy; ^2^Department of Life and Health Sciences, Link University, Via del Casale di S. Pio V, 44, 00165, Rome, Italy; ^3^Institute for Biomedical Research and Innovation (IRIB), National Research Council of Italy (CNR), Messina, Italy; ^4^Behavioral Neuroscience Lab, Champlain College, Burlington, VT, United States; ^5^Department of Psychology and Education, Kharazmi University, Tehran, Iran

**Keywords:** new technologies, neurodevelopmental rehabilitation, neurodevelopmental disorders (NDDs), cognitive training, motor training, cognitive processes

The use of new technologies in neurodevelopmental rehabilitation has gained increased interest over the last decades. Several new rehabilitation approaches that use technologies in different way, ranging from neurofeedback, to telerehabilitation, to computer-based *ad-hoc* interventions, have been used in this specific field and proved to be useful (Damianidou et al., 2018; Lancioni, 2018).

Following on the first promising results reported in the literature, this Research Topic aimed to explore recent developments in this area with a focus on psychological and technological research that investigated how new technological interventions for neurodevelopmental rehabilitation can offer a better opportunity to treat cognitive, motor, and social deficits in children with Neurodevelopmental Disorders (NDDs).

A first study (Cancer et al.) applied neurofeedback (NF) with the aim of modulating the inter-hemispheric balance of the temporal–parietal regions in developmental dyslexia (DD). In this specific study, NF training focused on reading skills was applied to 40 adults with a diagnosis if DD. The study also included a control condition which used sham NF, which is something often lacking in similar research studies. The NF experimental intervention consisted of three NF sessions (warm up, training and reinforcement) that were based on the use of the ProComp5 Infiniti software paired with the BioGraph Infiniti Software. This study showed a significant effect of the NF based intervention to improve reading abilities in adults with DD.

A second study of Valle et al., proposed a new perspective on the role of self-confidence and confidence in the evaluation and rehabilitation of children with adverse childhood experience (ACE) and borderline intellectual functioning (BIF). The authors investigated the characteristics of the internal working models of these children by applying the separation anxiety test, using both the classical and a new coding system to identify the specific features of the attachment representation. Results indicated that these children showed low self-confidence and high separation anxiety, with a tendency to somatization. In this case the focus on technology was more indirect, if extremely relevant, since the authors focused on the role that the attachment profile has on the efficacy of the use of technological devices in the rehabilitation process. The results from this paper highlight how it is necessary to consider the attachment representation of children with ACE of BIF as a significant variable affecting technological interventions combined with cognitive and behavioral variables.

Another study of Lecciso et al. focused on emotions, by exploring the effectiveness of two technological-based interventions in improving the expression of basic emotions in children with autism spectrum disorder (ASD). The authors conducted a pre-post study comparing a robot-based type of training with a computer-based type of training. Twelve children with ASD, aged from 6 to 13 years, were randomly assigned into two groups (robot vs. computer intervention). Both interventions showed an improved in children's ability to express emotions, and no significant difference between the two types of intervention was found. The authors argued that technological interventions, regardless of type of devices, can be consider a valid tool for a neurodevelopmental rehabilitation focused on improving the expression of emotions when social skills are impaired.

Two studies included in this Research Topic focused on the effectiveness of telerehabilitation (TR). Menici et al. presented a case study in which a 17-year-old female with a motor disorder received a Virtual Reality Rehabilitation System (VRRS) HomeKit, developed by Khymeia. The treatment was carried out at home, the therapist checked the performance of patient in different motor tasks through a platform. Results indicated an improvement of motor skills, suggesting both the effectiveness of the TR and the use of VRRS HomeKit. Lotan et al. presented a study on the effects of a skype-based, telehealth-delivered physical activity program carried out by participants' parents at home, showing similar positive results.

In the last study included in this Research Topic, 40 participants with Rett Syndrome (RTT), aged between from 10 to 25 years, and their families were involved in a 12-week individualized daily physical activity program. The performance was evaluated three times through skype meeting with the therapist. Results indicated an improvement of participants' motor skills and user satisfaction, suggesting a positive impact of TR.

The present Research Topic gives an overview about the current rehabilitation approaches using new technologies in the NDDs. All studies included in this Research Topic show positive results of technological interventions in different NDDs. However, due to the heterogeneity of NDDs, to design a technological intervention in this area, we highlight that it is necessary: (a) to consider the specific clinical features of disorder treated; (b) to include both psychological, cognitive and behavioral variables; (c) to involve a control group or condition. Based on these findings, we suggest that the use of new technologies can be a valid rehabilitation approach that does not replace a traditional intervention but can maximize the benefits of it.

## Author Contributions

TC and RF drafted a first version of this Editorial. All authors contributed to and approved the final version.

## Conflict of Interest

The authors declare that the research was conducted in the absence of any commercial or financial relationships that could be construed as a potential conflict of interest.

## Publisher's Note

All claims expressed in this article are solely those of the authors and do not necessarily represent those of their affiliated organizations, or those of the publisher, the editors and the reviewers. Any product that may be evaluated in this article, or claim that may be made by its manufacturer, is not guaranteed or endorsed by the publisher.
